# Role of transgelin-2 in diabetes-associated pancreatic ductal adenocarcinoma

**DOI:** 10.18632/oncotarget.17519

**Published:** 2017-04-29

**Authors:** Yan Sun, Weiwei He, Man Luo, Yuhong Zhou, Guilin Chang, Weiying Ren, Kefen Wu, Xi Li, Jiping Shen, Xiaoping Zhao, Yu Hu

**Affiliations:** ^1^ Department of Geriatrics, Zhongshan Hospital, Fudan University, Shanghai 200032, China; ^2^ Department of Thoracic Surgery, Sixth People's Hospital, School of Medicine, Shanghai Jiao Tong University, Shanghai 200025, China; ^3^ Department of Oncology, Zhongshan Hospital, Fudan University, Shanghai 200032, China; ^4^ Department of Nuclear Medicine, Ren Ji Hospital, School of Medicine, Shanghai Jiao Tong University, Shanghai 200025, China

**Keywords:** transgelin-2, SREBP, PDAC, diabetes, insulin

## Abstract

Pancreatic ductal adenocarcinoma (PDAC) is an aggressive malignancy with poor prognosis. Diabetes is a significant risk factor for PDAC and >50% of PDAC patients have concomitant diabetes. How diabetes influences the initiation and progression of PDAC remains elusive. Here, we show that transgelin-2 is dominantly expressed in PDAC tissues compared with adjacent normal tissues. The high level of transgelin-2 indicates poor survival of patients with PDAC. Remarkably, transgelin-2 expression is correlated with diabetic status. Hyperinsulinemia is frequently observed in type 2 diabetes. Our results indicate that upregulation of transgelin-2 is induced by insulin via sterol regulatory element-binding protein (SREBP)-1-mediated transcription in PDAC cells. Transgelin-2 is a novel target of SREBP-1. Our data support a novel mechanism in diabetes-associated PDAC by which transgelin-2 mediates proliferation of PDAC cells upon insulin stimulation. The insulin/SREBP-1/transgelin-2 network should be further explored as a diagnostic marker or a novel therapeutic target for diabetes-associated PDAC.

## INTRODUCTION

Approximately 95% of pancreatic cancers are pancreatic ductal adenocarcinoma (PDAC). The average 5-year survival rate is only 8% [1]. Reasons for the dismal survival include an aggressive disease course, low rate of early diagnosis, and limited understanding of the disease etiology. Several lines of evidence suggest that diabetes plays a critical role in the pathogenesis of PDAC. Elevated insulin level is frequently observed in type 2 diabetes, which makes up ∼90% of cases of diabetes. Given that insulin is a powerful mitogen, a sustained increase of insulin may confer growth advantages upon pancreatic cells [2]. It is therefore imperative to investigate the effects of insulin on pancreatic cancer progression.

Transgelin-2, encoded by *TAGLN2* gene, is an actin stress fiber-associated protein that has roles in cell transformation and cell morphology. Several proteomic studies have suggested that transgelin-2 is a potential biomarker of tumorigenesis. Transgelin-2 is overexpressed in colorectal cancer, renal cell carcinoma and uterine cervical squamous cell carcinoma [3–5]. In contrast, some reports indicate that transgelin-2 is downregulated in lung adenocarcinoma and breast cancer [6, 7]. Through regulating cytoskeletal dynamics, transgelin-2 also participates in cancer metastasis [8]. These studies have established the connection between transgelin-2 and cancer. Both oncogenic and tumor suppressive effects of transgelin-2 have been observed depending on the types of tissues investigated. However, with regard to PDAC, the expression pattern and functional role of transgelin-2 have not been determined.

Sterol regulatory element-binding proteins (SREBPs) are master regulators of genes for central rate-limiting enzymes of lipid and cholesterol metabolism. SREBP transcription factors regulate several enzymes including fatty acid synthase (FASN), acetyl-CoA carboxylase (ACC), stearoyl-CoA desaturase (SCD)-1, ATP citrate lyase (ACLY) and acyl-CoA synthetase (ACS)-2 [9, 10]. Mammalian SREBPs are encoded by the genes *SREBF1* and *SREBF2*, which are mainly involved in lipid and cholesterol metabolism, respectively. SREBP-1 knockdown decreases the cell and organ size of flies, indicating that it is essential for cell growth [11]. Pharmacological inhibition of SREBP-1 significantly induces cancer cell death [12].

The goal of this study was to characterize the expression pattern of transgelin-2 in PDAC and subsequently unravel the underlying mechanisms involved in dysregulation of transgelin-2. Our data indicated that transgelin-2 was highly expressed in PDAC tissues compared with adjacent normal tissues. Transgelin-2 was predominantly overexpressed in a subgroup of PDAC patients with diabetes. *In vitro* analysis indicated that insulin was a driving factor for expression of transgelin-2 via SREBP-1 transcription factor. These data imply that transgelin-2 is involved in progression of PDAC patients with concomitant diabetes. Diabetes is a key risk factor of PDAC, therefore, understanding the underlying mechanisms involved in diabetes-associated PDAC is important in providing novel biomarkers or therapeutic targets for this type of malignancy.

## RESULTS

### Upregulated transgelin-2 is an indicator of poor prognosis of PDAC

**Figure 1:**
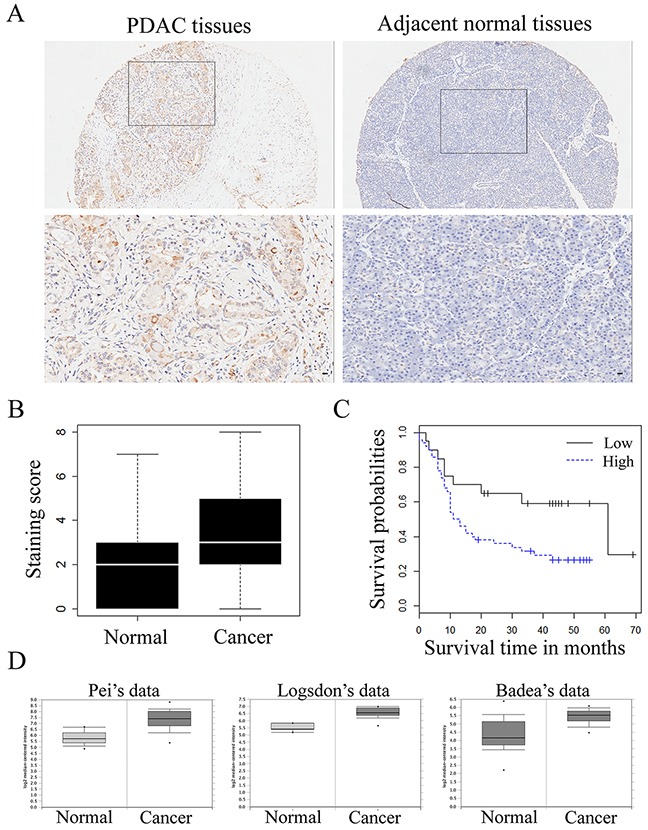
Transgelin-2 expression in PDAC and adjacent normal tissues **(A)** Representative pictures of transgelin-2 immunostaining in PDAC (left) and adjacent normal (right) tissues. Location of the high magnification regions (upper panels) is indicated by the rectangles in the upper panels. Magnified images of cells counterstained with transgelin-2 antibody are shown in lower panel. Scale bar, 100 μm. **(B)** Mean staining score of transgelin-2 in PDAC tissues compared with and adjacent normal tissues. **(C)** Overall survival as a function of transgelin-2 expression. High levels of transgelin-2 was labeled as blue, while low levels of transgelin-2 was labeled as black. Analysis of statistical significance was performed using the long-rank test. **(D)** Transgelin-2 level in pancreatic cancer tissues and corresponding normal tissues in Pei's dataset (p<0.01), Logsdon's dataset (p<0.01) and Badea's dataset (p<0.01).

To analyze the role of transgelin-2 in PDAC, we examined its protein level by immunohistochemical staining in a sample of 70 paired PDAC and adjacent normal tissues. Characteristics of the 70 PDAC cases are shown in Table 1. Immunostaining of transgelin-2 was mainly distributed in the cytoplasm, as shown by the representative images of IHC staining for transgelin2 (Figure 1A). The intensity of immunostaining for transgelin-2 was much stronger in PDAC tissues, while the adjacent normal tissues displayed negative or weak staining (Figure 1B). Semi-quantitative immunohistochemical analysis indicated that transgelin-2 was highly expressed in 71% of PDAC tissues and 30% of adjacent normal tissues. The level of transgelin-2 was significantly higher in PDAC tissues compared with normal tissues (p=3.647×10^−8^, Figure 1B). Furthermore, Pei's dataset (p=2.35×10^−10^), Logsdon's dataset (p=1.71×10^−5^) and Badea's dataset (p=1.36×10^−9^) consistently showed that transgelin-2 expression was significantly higher in pancreatic tissues than normal tissues (Figure 1D) [13–15]. Using a Cox proportional hazard regression model, high levels of transgelin-2 were associated with poor survival of PDAC patients (hazard ratio=2.30, 95% confidence interval: 1.07–4.97, p<0.05). The median overall survival was significantly lower in patients with high transgelin-2 expression (Figure 1C).

**Table 1:** Correlation between transgelin-2 level and clinicopathological parameters of patients with PDAC

Characteristics	All cases	Transgelin-2		*p* value
		Low	High	
Total participants	70	20	50	
Age (yr)				
<60	27	5	22	0.229
≥60	43	15	28	
Gender				
Female	23	5	18	0.546
Male	47	15	32	
Histological grade				
Well	4	3	1	0.043
Moderately	50	15	35	
Poorly	16	2	14	
Tumor stage				
1A	3	2	1	0.051
1B	25	10	15	
2A	7	2	5	
2B	34	5	29	
4	1	1	0	
Primary tumor				
T1	4	2	2	0.591
T2	53	14	39	
T3	13	4	9	
Lymph node metastasis				
Negative	36	15	21	0.027
Positive	34	5	29	
Tumor size				
≤2 cm	23	11	12	0.027
>2 cm	47	9	38	
Diabetes				
Without	29	13	16	0.024
With	41	7	34	

The clinicopathological characteristics of PDAC stratified by the extent of transgelin-2 expression are summarized in Table 1. The level of transgelin-2 was significantly associated with lymph node metastasis, histological grade, and tumor stage and size. However, there was no correlation with patient age, gender and primary tumor. PDAC patients with diabetes showed high expression of transgelin-2 compared with those without diabetes. Among these factors, histological grade (odds ratio [OR] =5.293, p=0.030), tumor size (OR=5.357, p=0.017) and diabetes (OR=4.623, p=0.020) were proven to be independent predictors of high transgelin-2 expression by multivariate logistic regression analysis (Table 2). These results prompted us to investigate the role of transgelin-2 in diabetes-associated PDAC.

**Table 2:** Factors correlated to transgelin-2 levels by multivariate logistic analysis

Factor	Odds ratio	*p* value
Histologic grade	5.293	0.030
Lymph node metastasis	2.087	0.274
Tumor size	5.357	0.017
Diabetes	4.623	0.020

### Insulin increases expression of transgelin-2 in PDAC cells

**Figure 2:**
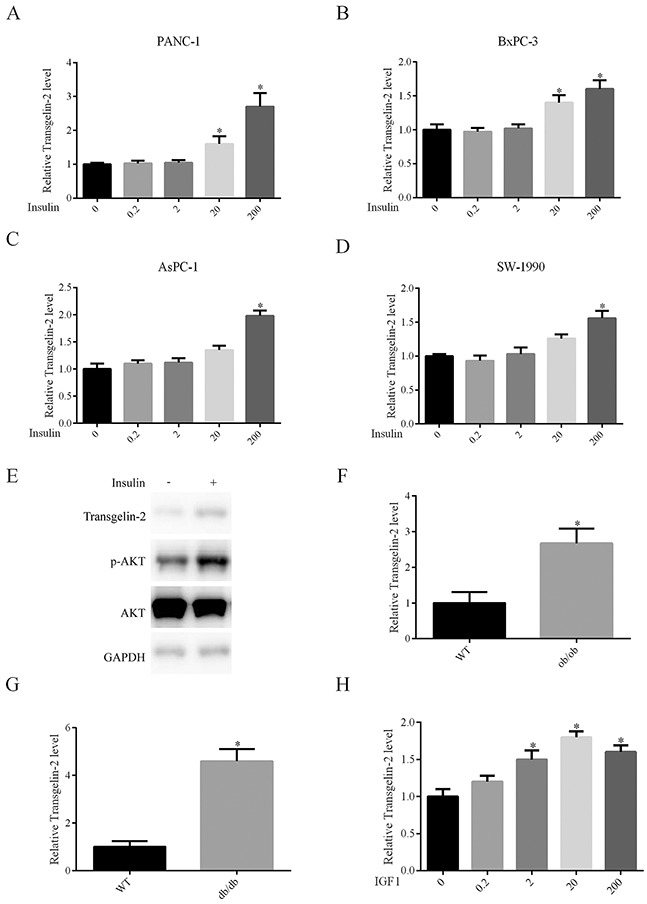
Impact of insulin on transgelin-2 expression PANC-1 **(A)**, BxPC-3 **(B)**, AsPC-1 **(C)** and SW-1990 **(D)** cells were treated with insulin at dose 0, 0.2, 2, 20 and 200 nM for 24 h, respectively. Transgelin-2 was detected by quantitative PCR. The relative transgelin-2 level was quantified by 2^−ΔΔCT^ method, with GAPDH as a reference gene. **(E)** PANC-1 cells were treated with 200 nM insulin for 24 h. Transgelin-2 level, AKT level and AKT S473 phosphorylation were analyzed by western blotting. Quantitative PCR analysis of transgelin-2 mRNA in pancreatic tissues from ob/ob **(F)** or db/db **(G)** mice demonstrating increased transgelin-2 in type 2 diabetes models. **(H)** Quantitative PCR analysis displaying increased transgelin-2 mRNA by IGF-1 in a dose-dependent manner. In all cases, data are represented as mean±SD of three independent experiments.

Type 2 diabetes is characterized by high levels of insulin, which are presumed to be a driving factor of diabetes-associated cancer [16, 17]. Thus, we hypothesized that insulin might be involved in regulation of transgelin-2 in PDAC. To test this hypothesis, PANC-1 cells were treated with insulin for 24 h. Insulin treatment resulted in an increase in transgelin-2 mRNA level in a dose-dependent manner (Figure 2A). Similar to PANC-1, PDAC cell lines AsPC-1, BxPC-3 and SW-1990 also exhibited upregulation of transgelin-2 upon insulin treatment (Figure 2B–2D). Insulin treatment for 24 h resulted in marked accumulation of transgelin-2 protein (Figure 2E). The insulin signaling pathway, as indicated by AKT phosphorylation (S473), was consistently activated by insulin treatment in PANC1 cells (Figure 2E) [18]. We studied the expression profile of transgelin-2 in both db/db and ob/ob mouse type 2 diabetes model, which display a hyperinsulinemia phenotype [19]. Transgelin-2 was significantly up-regulated in pancreatic tissues from both db/db (Figure 2F) and ob/ob (Figure 2G) mice. These *in vitro* and *in vivo* data suggest that expression of transgelin-2 is under-regulated by insulin. Insulin and insulin-like growth factor (IGF)-1 have affinity for insulin and IGF-1 receptors because of similar structural homology [20]. Thus, we also checked the levels of transgelin-2 upon IGF-1 treatment. IGF-1 also increased the levels of transgelin-2 in PANC1 cells (Figure 2H). This suggests that downstream effectors of insulin or IGF-1 mediate increased transgelin-2 expression in PDAC cells.

### Transgelin-2 is required for insulin-induced proliferation of PDAC cells

**Figure 3:**
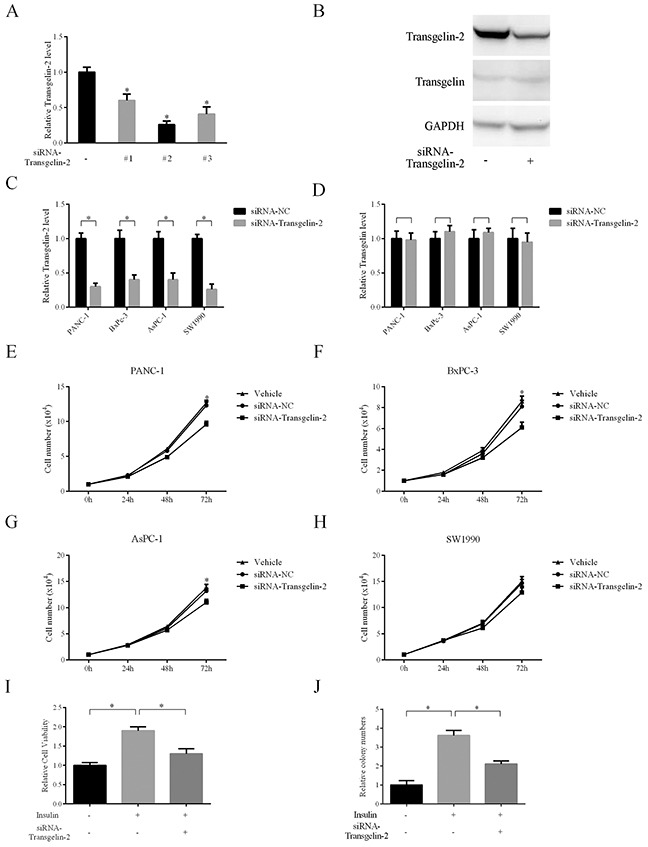
Impact of transgelin-2 depletion on proliferation of PDAC cells **(A)** Three specific siRNAs against transgelin-2 were transfected into PANC-1 cells for 48 h. The knockdown efficiency was analyzed by quantitative PCR. **(B)** Western blot analysis of transgelin-2 or transgelin protein level in PANC-1 cells after transfection for 48 h. PDAC cell lines (PANC-1, AsPC-1, BxPC-3 and SW-1990) were transfected by specific siRNAs against transgelin-2 for 48 h. Transgelin-2 **(C)** or transgelin **(D)** mRNA level was analyzed by quantitative PCR. Cell proliferation was analyzed in PANC-1 **(E)**, AsPC-1 **(F)**, BxPC-3 **(G)** and SW-1990 **(H)** cell lines treated with non-silencing siRNA (siRNA-NC) or specific siRNA against transgelin-2. Vehicle group indicates cells treated with transfection reagent alone. **(I)** Relative cell viability was analyzed in PANC-1 cells treated with siRNA against transgelin-2 in the presence or absence of insulin. **(J)** Colony formation was performed in PANC-1 cells with siRNA against transgelin-2 in the presence or absence of insulin. In all cases, data are represented as mean±SD of three independent experiments.

To elucidate the oncogenic role of transgelin-2 in PDAC, cell proliferation was analyzed when transgelin-2 was silenced in PDAC cells. Of the three specific siRNAs against transgelin-2, transgelin2-siRNA-2 displayed the most significant knockdown effect (Figure 3A). The protein level of transgelin-2 was also decreased by transgelin2-siRNA-2, while transgelin level, a homolog of transgelin-2, was unaffected by this siRNA (Figure 3B). Therefore, transgelin2-siRNA-2 was used as a specific siRNA against transgelin-2 in the subsequent experiments. In a panel of PDAC cells, transgelin-2 but not transgelin was decreased by this siRNA (Figure 3C and 3D). Transgelin-2 knockdown decreased proliferation of PANC-1 cells (Figure 3E). Similarly, the growth of BxPC-3 and AsPC-1 cells was reduced by transgelin-2 knockdown (Figure 3F and 3G). There was no significant inhibitory effect of transgelin-2 knockdown on growth of SW1990 cells (Figure 3H). This suggests that transgelin-2 may have distinct roles with different genetic backgrounds. More importantly, transgelin-2 knockdown inhibited proliferation of PANC-1 cells when they were treated with insulin (Figure 3I). The colony-forming ability induced by insulin was blunted by transgelin-2 knockdown in PANC1 cells (Figure 3J). These data suggest that oncogenic effects of insulin are likely due to the increase of transgelin-2 in PDAC.

### SREBP-1 mediates increase of transgelin-2 expression induced by insulin

**Figure 4:**
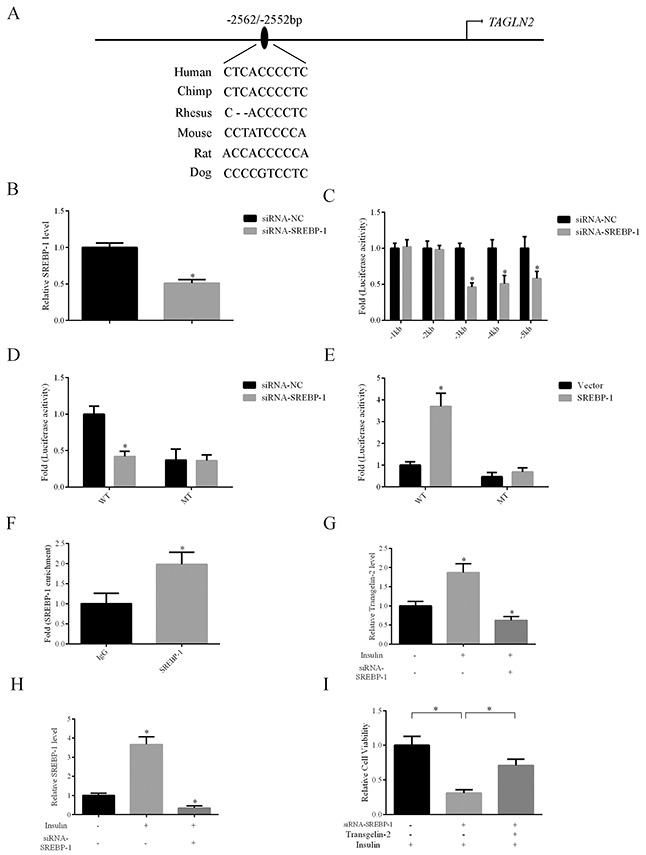
Transgelin-2 is a target gene of SREBP-1 **(A)** Schematic diagram of promoter region of the human *TALGN2* gene. A putative SRE site is located at −2562/−2552 bp. **(B)** Validation of SREBP-1 knockdown efficiency using quantitative PCR. **(C)** Luciferase activity assay of transgelin-2 reporter containing different fragments spanning the promoter region, indicating that the SRE sequence was located between 1 and 5 kb. **(D)** HEK293T cells were co-transfected by non-silencing siRNA (siRNA-NC) or siRNA against SREBP-1 (siRNA-SREBP-1) with wild-type (WT) or mutant (MT) transgelin-2 firefly luciferase reporter for 24 h. Luciferase activity was measured. The fold of luciferase activity was calculated by normalization of the firefly luciferase activity by the transfection containing reporter plasmids and the siRNA-NC. **(E)** HEK293T cells were co-transfected by vector or SREBP-1 with wild-type (WT) or mutant (MT) transgelin-2 firefly luciferase reporter for 24 h. The fold of luciferase activity was calculated by normalization of the firefly luciferase activity by the transfection containing reporter plasmids and the siRNA-NC. **(F)** ChIP analysis using SREBP-1 antibody demonstrated SREBP-1 association with SRE sequence within transgelin-2 promoter. **(G)** Quantitative PCR analysis of transgelin-2 mRNA in PANC-1 cells treated with or without 200 nM insulin for 24 h. **(H)** Quantitative PCR analysis of SREBP-1 mRNA in PANC-1 cells treated with or without 200 nM insulin for 24 h. **(I)** Proliferation was analyzed in PANC-1 cell lines transfected by siRNA-SREBP-1 (siRNA-NC as a control) with or without transgelin-2 in the presence of insulin. In all cases, data are represented as mean±SD of three independent experiments.

Since the expression of transgelin-2 is induced by insulin, we explored whether the insulin-related transcription factor is responsible for the increase of transgelin-2 in PDAC. Using the JASPAR database [21], we scanned 5 kb prior to the human *TAGLN2* transcription start site at an 85% profile score threshold. A putative SRE site for SREBP-1 transcription factor was found at −2562/−2552 bp on the human *TAGLN2* promoter (Figure 4A). To study the responsiveness of the putative SRE site upon SREBP-1 knockdown, we constructed a series of reporter vectors containing fragments of transgelin-2 promoter. Endogenous SREBP-1 was downregulated by siRNA against *srebf1* gene in HEK293T cells (Figure 4B). Analysis of promoter fragments indicated that the putative SREBP-1 binding site was present at approximatively from −2000 to −5000 bp on *TAGLN2* promoter, in line with our *in silico* assay (Figure 4C). To ascertain the functionality of the putative SRE site on *TAGLN2* gene, luciferase reporter vectors containing wild-type or mutant SRE sequences were transfected into HEK293T cells. Luciferase assay showed that the wild-type but not mutant luciferase activity was responsive upon SREBP-1 knockdown (Figure 4D). Conversely, overexpression of SREBP-1 significantly increased the firefly luciferase activity of wild-type but not mutant transgelin-2 luciferase reporter (Figure 4E). *TAGLN2* gene regulatory elements at −2562/−2552 bp were enriched for SREBP-1 antibody when compared with an IgG-negative control, suggesting that this locus is a potential SREBP-1 target (Figure 4F). Our data support a notion that *TAGLN2* is a novel target gene of the SREBP-1 transcription factor. In addition, SREBP-1 knockdown blunted the increase of transgelin-2 expression induced by insulin in PANC-1 cells (Figure 4G). SREBP-1 was consistently induced by insulin, whereas the specific siRNA downregulated its level (Figure 4H). These results indicate that SREBP-1 is involved in activating transgelin-2 transcription upon insulin stimulation. Moreover, transgelin-2 partially rescued the growth defect of pancreatic cancer cells caused by SREBP-1 knockdown, suggesting that transgelin-2 is required for SREBP-1-mediated cell growth upon insulin treatment (Figure 4I).

### Transgelin-2 and SREBP-1 are correlated in diabetes-associated PDAC tissues

**Figure 5:**
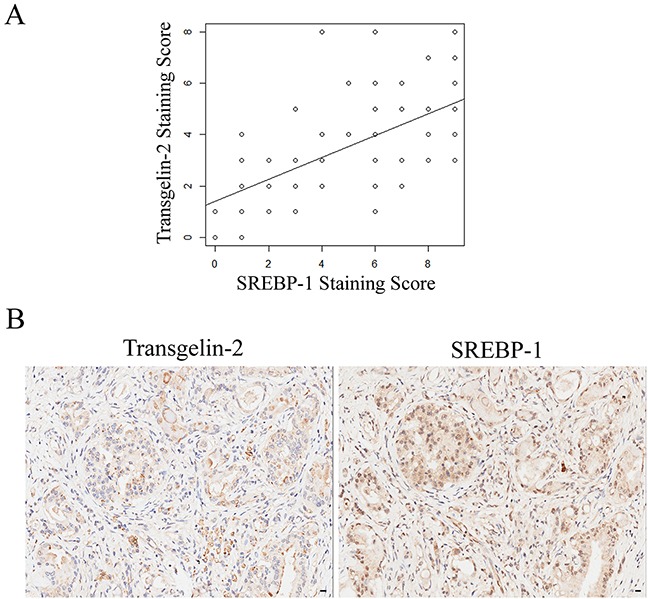
Correlation between transgelin-2 and SREBP-1 **(A)** Scatter plot between transgelin-2 and SREBP-1 staining scores in a sample of PDAC patients. The correlation analysis was calculated using Spearman statistical analysis (Spearman's correlation coefficient: 0.617, p<0.001). R^2^ is 0.3807. **(B)** Representative photomicrographs of transgelin-2 (left) and SREBP-1 (right) staining in PDAC tissues from the same patient.

We investigated whether transgelin-2 and SREBP-1 were correlated in diabetes-associated PDAC. SREBP-1 expression was also analyzed by immunohistochemistry in tissues from PDAC patients, as described previously (Table 1). The staining for transgelin-2 or SREBP-1 was quantitated according to staining intensity and percentage of positive cells. Spearman statistical analysis was performed to assess the relationship between transgelin-2 and SREBP-1. Transgelin-2 was highly correlated with SREBP-1 in PDAC tissues (Spearman's correlation coefficient: 0.617, p<0.001) (Figure 5A). Concomitant with the high SREBP-1 expression, we noted an increase in transgelin-2 expression (Figure 5B). According to diabetic status, we stratified PDAC patients into two groups. PDAC tissues showed high expression of transgelin-2 and SREBP-1, which were mainly distributed in patients with diabetes (Table 3). The χ^2^ test demonstrated that the levels of transgelin-2 and SREBP-1 were correlated in PDAC patients with diabetes compared to those without diabetes. These data suggest that the insulin/SREBP-1/transgelin-2 signaling axis is involved in diabetes-associated PDAC.

**Table 3:** Correlation between transgelin-2 and SREBP-1 as stratified by diabetic status in PDAC patients

		Without diabetes			With diabetes		
		Transgelin-2		*p* value	Transgelin-2		*p* value
		Low	High		Low	High	
SREBP-1	Low	10	6	0.081	6	1	<0.001
	High	3	10		1	33	

## DISCUSSION

Diabetes is typically divided into type 1 and type 2. Type 2 diabetes mellitus (T2DM) comprises >90% of all cases of diabetes. T2DM consists of an array of dysfunctions characterized by hyperglycemia, which results from insulin resistance, inadequate insulin secretion, and excessive glucagon secretion. Epidemiological studies clearly indicate that diabetes (predominantly T2DM) is a risk factor for liver, pancreatic and endometrial cancer. Nearly half of PDAC patients have diabetes at the time of diagnosis. Here, we found that transgelin-2 is dominantly expressed in PDAC patients with diabetes, which suggests that transgelin-2 is involved in progression of diabetes-associated PDAC. A homolog of transgelin-2, transgelin, is also highly expressed in PDAC patients with diabetes [22]. We presume that transgelin-2 and its homolog are extensively involved in promoting diabetes-associated PDAC. In this study, we focused on transgelin-2 to decipher the underlying mechanisms that mediate its upregulation in diabetes-associated PDAC.

It remains unclear whether the association between PDAC and T2DM is direct or indirect [23]. The direct effect is probably due to hyperglycemia, hyperinsulinemia and insulin resistance. Insulin is produced by pancreatic β cells. In the case of T2DM, pancreatic cells are persistently exposed to high concentrations of endogenous insulin, which provides a hospitable environment for tumorigenesis [24]. Insulin is a growth-promoting hormone with mitogenic effects. PDAC cells commonly overexpress insulin and IGF-1 receptors, which play a key role in cell growth and differentiation. Hyperinsulinemia is viewed as an independent risk factor of PDAC [25]. Therefore, the casual nature of the association between diabetes and PDAC is largely due to hyperinsulinemia [26]. The molecular mechanism of hyperinsulinemia-related proliferation and tumorigenesis has not been fully elucidated. In the present study, our data indicate that transgelin-2 is induced by insulin in PDAC cells, and the effect of insulin on the growth of PDAC cells is dependent on transgelin-2. These data suggest that induction of transgelin-2 by insulin participates in the development of diabetes-associated PDAC. Thus, blocking transgelin-2 expression is a potential therapeutic strategy to treat PDAC occurring concomitantly with diabetes. It also raises an interesting question about the role of transgelin-2 in initiating tumorigenesis in diabetes-associated PDAC. In the present study, the association of transgelin-2 expression with diabetic status in normal tissues was unknown. It would be worthwhile to analyze transgelin-2 levels in normal pancreatic tissues prior to malignant transformation in a future study.

Multiple signaling pathways are activated after insulin and IGF-1 interacts with their receptors. Several studies have demonstrated that the PI3K/AKT and RAS/MEK/ERK pathways are extensively stimulated by insulin in PDAC cells. Once activated, these signaling pathways stimulate multiple cancer phenotypes, including proliferation, invasion and metastasis. Besides the direct effects of insulin on PDAC cells, insulin can increase the level of IGF-1 via decreasing the hepatic production of IGF binding proteins [27]. It is well established that transcriptional activity of SREBP-1 is induced by insulin signaling [28]. Upon insulin stimulation, SREBP-1 is transported to the Golgi apparatus where it is cleaved by S1P and S2P proteases and then shuttled to the nucleus to induce expression of target genes. SREBP-1 target genes including *FASN*, *SCD1* and *ACLY* are mainly involved in lipid biosynthesis. We have previously identified that regulation of SREBP-1 activity by cyclin-dependent kinase 8 and its partner cyclin C is involved in insulin-induced triglyceride synthesis [9]. Here, we found that transgelin-2 is a novel target gene of SREBP-1 transcription factor in response to insulin stimulation. In diabetes-associated PDAC, hyperinsulinemia promotes the maturation and transcriptional activity of SREBP-1, which leads to an increase of transgelin-2. Thus, PDAC tissues from patients with diabetes display a high level of transgelin-2. The axis of insulin/SREBP-1/transgelin-2 links the association between diabetes and PDAC, suggesting that this signaling pathway represents new therapeutic targets and molecular markers for this subgroup of PDAC. Further studies are also required to analyze the biological function of transgelin-2 in diabetes-associated PDAC.

Several studies have demonstrated that the level of transgelin-2 is associated with prognosis in various cancers. Transgelin-2 has been linked to lymph node metastasis, advanced clinical stage, and survival of cancer patients. In the present study, lymph node metastasis, histological grade, and tumor stage and size were closely related to the level of transgelin-2 in PDAC. Upregulation of transgelin-2 predicts poor survival of patients with PDAC, and demonstrates that transgelin-2 is involved in cancer progression. Transgelin-2 may be a useful biomarker for diagnosis or evaluation in PDAC. Leptin-deficient ob/ob and leptin-receptor-deficient db/db mice are widely used for T2DM research. Hyperinsulinemia is a common characteristic of these two models. Of note, transgelin-2 transcription is also activated in mouse diabetic models. The expression pattern of transgelin-2 is unable to distinguish between diabetes and PDAC.

Perhaps because of its abundance, transgelin-2 has been frequently identified in proteomic profiling studies of cancer [29]. The underlying mechanisms of transgelin-2 in cancer behavior is involved in interacting with specific proteins. For example, transgelin-2 regulates metastasis via directly interacting with actin and changing cell motility. In other cases, transgelin-2 influences signaling pathways through association with cancer-related proteins. It is worth further exploring the detailed molecular mechanisms of transgelin-2 in tumorigenesis and progression of cancer. Several studies have demonstrated that transgelin-2 is dysregulated in various types of cancer. However, until now, its transcriptional regulation remained unstudied. The present study indicated that SREBP-1 is responsible for transcription of transgelin-2 upon insulin treatment in PDAC cells. Luciferase reporter and chromatin immunoprecipitation (ChIP) analysis have shown that transgelin-2 is a novel target of SREBP-1, which is induced by insulin. These data explain why transgelin-2 is upregulated in diabetes-associated cancer.

## MATERIALS AND METHODS

### Patients and tissue specimens

This work was done with the approval of the Ethics Committee of Zhongshan Hospital, and informed consent was obtained from all patients. Formalin-fixed paraffin-embedded tissue blocks containing 70 PDAC and 70 adjacent tumor-free tissues were subjected to immunostaining. All of the patients underwent pancreatectomy between September 2004 and March 2009. There were 38 male and 22 female patients. The median follow-up was 16 months (range, 0–69 months), and 45 of 70 patients died during follow-up. Patients who did not reach the outcome under study were censored at the date of their last visit. For the analyses of overall survival, each patient's time began on the date of diagnosis and ended on the date of death or on the date last seen alive.

### Cell lines and transfection

PANC-1, AsPC-1, BxPC-3, SW-1990 and HEK293T cell lines were purchased from ATCC (Manassas, VA, USA). Cells were maintained in DMEM or RPMI 1640 supplemented with 10% fetal calf serum and antibiotics (Invitrogen, Carlsbad, CA, USA) in a humidified atmosphere under 5% CO_<sub>2</sub>_. All transfections were performed with Lipofectamine 2000 (Thermo Scientific, Waltham, MA, USA) or Lipofectamine RNAiMAX (Thermo Scientific) transfection reagents. Cells were cultured until 40%–50% confluence at the time of transfection. At 24–48 h after transfection, cells were harvested for quantitative PCR or western blot analysis. For gene knockdown experiments, control cells were incubated with OPTI-MEM and transfection reagent (vehicle group), or with OPTI-MEM and transfection reagent plus no-silencing siRNA (siRNA-NC group). Sequences of siRNA were: transgelin-2#1, 5′-GGUUACAGAUGGGCACCAAUU-3′, transgelin-2 #2, 5′- CCUAAGAAAUCCAAGGAGAUU -3′, trans gelin-2#3, 5′- UCAAGCAGAUGGAGCAGAUUU -3′, SREBP-1, 5′- GGGAGAGCCUGUACAGCUUUU-3′.

### Luciferase activity assay

To construct the reporter plasmid of transgelin2-Luc, fragments of the promoter region of *TAGLN2* gene were amplified by PCR and cloned into pGL3 vector. HEK293T cells were co-transfected with 100 ng transgelin2-Luc reporter and 10 ng RGB renilla luciferase (as an internal control). After 24 h transfection, cells were harvested and washed with phosphate-buffered saline (PBS), and lysed on ice in passive lysis buffer. Reporter gene assays were performed with the Dual-Luciferase Reporter Assay System (Promega, Madison, WI, USA). All experiments were performed in triplicate from independent cell cultures.

### Immunohistochemical staining

Immunohistochemical staining of paraffin sections for transgelin-2 or SREBP-1 protein was performed with an LSAB kit (DAKO, Marseilles, France), using transgelin-2 antibody (dilution, 1:500; Novus Biologicals, Littleton, CO, USA) or SREBP-1 antibody (dilution, 1:250; Santa Cruz Biotechnology, Santa Cruz, CA, USA). The sections were incubated in 3,3′ diaminobenzide tetrahydrochloride with 0.05 % H_2_O_2_ for 3 min. Immunostaining scores were independently evaluated by three pathologists. Semi-quantitative scores were used to analyze antibody immunostaining. Intensity of staining was categorized into −, +, ++ or +++, denoting negative (0), weak (1), moderate (2) or strong staining (3). Extent of immunostaining was categorized into 0 (<10%), 1 (10%–25%), 2 (26%–50%) or 3 (>50%) on the basis of the percentage of positive cells. Three random microscopic fields per tissue were calculated. The final score of expression level was determined by the formula: final score = intensity score × percentage score. The final score was ranged from 0 to 9. The final score of ≤3 was defined as low expression, and >3 as high expression.

### Quantitative RT-PCR

To isolate total RNA, an RNeasy RNA isolation kit (Qiagen, Valencia, CA, USA) was used. The concentration of each RNA sample was measured using the Nanodrop Spectrophotometer (Thermo Scientific). The ratio of absorbance at 260 nm and 280 nm was used to assess RNA purity. The ratio for pure RNA was ∼2.0. Prior to cDNA synthesis, DNase treatment was performed using the RQ1 RNase-free DNase (Promega). RNA was subjected to cDNA synthesis (GE Healthcare, Marlborough, MA, USA). Real-time quantitative PCR was performed using the SYBR-Green Master PCR Mix kit (Thermo Scientific). Expression of mRNA was assessed by evaluating threshold cycle (CT) values. The comparative Ct (ΔΔCt) method was used to determine the relative mRNA level as described previously [30].. The primer sequences were as follows: transgelin-2 forward: GGAGATCTCTCCCCGCA, reverse TCCACTGGATCAGGATCTGC; SREBP-1 forward: GCTGCTGACCGACATCGAA, reverse GGGTGGGTCAAATAGGCCAG.

### Western blotting

Protein lysates were harvested from cells at 48 hours after transfection, washed with phosphate-buffered saline (PBS), and lysed on ice in lysis buffer (1% NP-40, 50 mM Tris–HCl pH 8.0, 150 mM NaCl and 2 mM EDTA) supplemented with complete protease inhibitor cocktail (Roche Applied Science, Indianapolis, IN, USA). The lysates were centrifuged at 15,000 rpm for 30 min at 4°C. The supernatant was collected. Protein concentration was quantitated by BCA method. About 30–50 μg of protein were separated by SDS−PAGE and then transferred to PVDF membranes. The membranes were probed with primary antibody for 3 h. After incubation with HRP-conjugated antibody, antibody detection was achieved by chemiluminescence. The primary antibodies used in this study were: as follow anti-transgelin-2 (Novus Biologicals), anti-transgelin (Abcam, Cambridge, MA, USA), anti-AKT (Santa Cruz Biotechnology), anti-phospho-AKT (Santa Cruz Biotechnology) and anti-GAPDH (Cell Signaling Technology, Danvers, MA, USA).

### Cell proliferation and colony-formation assay

Cell proliferation was performed as described previously [31]. Briefly, 10^4^ cells/well were seeded into six-well plates after 24 h transfection. Cell numbers were counted every 24 h. At least three independent experiments were performed. Growth curve assays were performed by counting live cells using trypan blue exclusion. For colony-formation assay, single-cell suspensions (500 cells per dish) were plated and fresh medium was replaced every 48 h. After 10–14 days, cells were fixed in methanol for 10 min, and stained with Crystal Violet (Sigma, St. Louis, MO, USA). Colonies were counted using an inverted microscope.

### ChIP assay

ChIP was performed using the EZ-ChIP Assay kit (Millipore, Billerica, MA, USA) with an antibody to SREBP-1 (Santa Cruz Biotechnology) as described previously [32]. Cells were grown overnight in 100-mm dishes to 60%–70% confluence (approximatively 0.8×10^7^−1.2×10^7^ cells/dish). Cells were crosslinked with 1% formaldehyde for 10 min and quenched with glycine for 5 min. Cells were harvested by scraping in ice-cold PBS and resuspended in SDS lysis buffer containing inhibitors (Roche Applied Science) and sonicated on ice to release 200–800-bp DNA fragments. The sheared DNA was incubated overnight with 10 μg SREBP-1 antibody with rotation at 4°C and precipitated with protein A/G agarose for 3 h at 4°C. Quantitative PCR was used to evaluate the enrichment of SREBP-1 on the promoter region of *TAGLN2* gene.

### Statistical analysis

Experimental results are presented as mean±SD. Wilcoxon test was used to assess the immunohistochemical score for transgelin-2 protein in cancer tissues and adjacent normal tissues. Correlation analysis of transgelin-2 staining score with clinicopathological features was analyzed by χ^2^ test. The correlation between transgelin-2 score and SREBP-1 score was examined by Spearman's test. For survival analysis, analysis of statistical significance was performed using the long-rank (Mantel–Cox) test. Multivariate logistic regression analysis was used for analyzing risk factors for the level of transgelin-2. Cox proportional hazard model was used to identify the prognostic factors for survival. A difference was considered significant at p<0.05. All statistical analysis was performed using SPSS software, version 22.
